# Avian disease surveillance on the island of San Cristóbal, Galápagos

**DOI:** 10.1002/ece3.8431

**Published:** 2021-12-06

**Authors:** Joshua G. Lynton‐Jenkins, Andrew F. Russell, Jaime Chaves, Camille Bonneaud

**Affiliations:** ^1^ Centre for Ecology and Conservation University of Exeter Penryn UK; ^2^ Department of Biology San Francisco State University San Francisco California USA; ^3^ Colegio de Ciencias Biológicas y Ambientales Universidad San Francisco de Quito Quito Ecuador

**Keywords:** avian pox, Avipoxvirus, El Nino, haemosporidia, small ground finch

## Abstract

Endemic island species face unprecedented threats, with many populations in decline or at risk of extinction. One important threat is the introduction of novel and potentially devastating diseases, made more pressing due to accelerating global connectivity, urban development, and climatic changes. In the Galápagos archipelago two important wildlife diseases: avian pox (*Avipoxvirus* spp.) and avian malaria (*Plasmodium* spp. and related Haemosporidia) challenge endemic species. San Cristóbal island has seen a paucity of disease surveillance in avian populations, despite the island's connectedness to the continent and the wider archipelago. To survey prevalence and better understand the dynamics of these two diseases on San Cristóbal, we captured 1205 birds of 11 species on the island between 2016 and 2020. Study sites included urban and rural lowland localities as well as rural highland sites in 2019. Of 995 blood samples screened for avian haemosporidia, none tested positive for infection. In contrast, evidence of past and active pox infection was observed in 97 birds and identified as strains Gal1 and Gal2. Active pox prevalence differed significantly with contemporary climatic conditions, being highest during El Niño events (~11% in 2016 and in 2019 versus <1% in the La Niña year of 2018). Pox prevalence was also higher at urban sites than rural (11% to 4%, in 2019) and prevalence varied between host species, ranging from 12% in medium ground finches (*Geospiza fortis*) to 4% in Yellow Warblers (*Setophaga petechial aureola*). In the most common infected species (Small Ground Finch: *Geospiza fuliginosa*), birds recovered from pox had significantly longer wings, which may suggest a selective cost to infection. These results illustrate the threat future climate changes and urbanization may present in influencing disease dynamics in the Galápagos, while also highlighting unknowns regarding species‐specific susceptibilities to avian pox and the transmission dynamics facilitating outbreaks within these iconic species.

## Graphical abstract

In the Galápagos archipelago, two important wildlife diseases, avian pox (*Avipoxvirus* spp.) and avian malaria (*Plasmodium* spp. and related Haemosporidia), challenge endemic species. We find avian pox prevalence differs significantly with prevailing climate, being highest during El Niño events (~11% in 2016 and in 2019 versus <1% in the La Niña year of 2018). Pox prevalence was also higher at urban sites than rural (11% to 4% in 2019), illustrating the threat future climate changes and urbanization may present in influencing disease dynamics in the Galápagos.

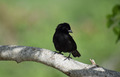

## 1 INTRODUCTION

Human activities are changing our planet's natural environment at an unprecedented rate and are driving the Earth's sixth mass extinction (Ceballos et al., 2015). Some contributors to population declines, such as habitat loss, are easily recognized, while others are less apparent. For instance, invasive species introduced to novel habitats can impact native species not only directly (e.g., through predation), but also indirectly (e.g., through competition) (Harris, 2009). Disease introductions can have particularly catastrophic consequences for island species, which typically have small populations that have been isolated from pathogen exposure for extensive periods of time (Wyatt et al., 2008). for instance, 41% of the endemic Hawai‘ian honeycreepers present when westerners arrived on the archipelago in the late 18th century have gone extinct (Atkinson & LaPointe, 2009; IUCN, 2021). Two introduced infectious diseases are thought to be largely responsible: avian malaria, caused by *Plasmodium relictum* and avian pox, caused by an *Avipoxvirus* (Atkinson & LaPointe, 2009). Islands are hotspots of species endemism and host a large proportion of global species richness (Kier et al., 2009). Conservation of global biodiversity will therefore benefit from a multi‐pronged approach towards the preservation of island species, encompassing not only habitat and species protections, but also disease management.

The Galápagos archipelago is a UNESCO world heritage site rich in avian species endemism. Birds in the Galápagos are confronted by a number of invasive species, including nest parasites (*Philornis downsii*; Fessl et al., 2001; McNew & Clayton, 2018) and avian nest predators (smooth‐billed ani (*Crotophaga ani*); Cooke et al., 2019). Avian diseases and their vectors (e.g., mosquitos) have also been introduced to the archipelago. Avian pox has afflicted endemic species on the islands since the late 19th century (Parker et al., 2011). Recent establishment of the mosquito *Culex quinquefasciatus*, a species known to feed on avian hosts, is also a concern. Populations of *C*. *quinquefasciatus* could not only alter the transmission dynamics of avian pox (as this species has been shown to feed on avian hosts more commonly than the endemic *Aedes taeniorhynchus*; Eastwood et al., 2019), but could also facilitate the introduction and establishment of virulent avian malaria parasites, for which it is a competent vector (*Plasmodium* spp.; Eastwood et al., 2019; LaPointe et al., 2012; Van Riper et al., 1986; Whiteman et al., 2005). While avian malaria has previously been detected in the Galápagos, prevalence has been low in avian populations (6% or less) and the virulent *Plasmodium relictum*, in part responsible for the decimation of the Hawai'ian avifauna, has not been detected (Levin et al., 2013; Valkiūnas et al., 2010). The threat of infectious disease to the Galápagos avifauna is made pressing by human population growth and high rates of visitation by tourists (Epler, 2007; Toral‐Granda et al., 2017). These factors both increase the likelihood of importing novel pathogens to the islands while also improving conditions for their establishment (e.g., by creating vector breeding sites; Louis et al., 2016). Active monitoring of avian populations for changes in infectious disease prevalence and the arrival of novel infectious pathogens is therefore critical to long‐term conservation.

San Cristóbal is the easternmost island of the archipelago and sits just over 900 km from continental South America. Puerto Baquerizo Moreno is situated in the west of the island; the provincial capital and second largest urban center in the Galápagos (INEC, 2015). Despite continuous habitation since the late 1800s, San Cristóbal has received considerably less disease surveillance by comparison with the archipelago's more centrally located and populous island of Santa Cruz, or compared with the more pristine islands of Floreana in the south, or Santiago and Isabela in the west (Asigau et al., 2017; Dudaniec et al., 2005; Jaramillo et al., 2017; Levin, 2013; Zylberberg et al., 2012, 2013). This represents a deficiency in our understanding of disease dynamics and emergence in Galápagos bird species for three reasons. First, San Cristóbal is well connected to the continent; both in terms of human activity (the town has both an airport and sea port), and as a stop‐over site for migrant bird species (Perlut & Renfrew, 2016). San Cristóbal also hosts an established population of the introduced *C*. *quinquefasciatus* mosquito, making the island a likely first‐contact zone for novel disease introductions (particularly avian malaria) (Whiteman et al., 2005). Second, the island is well connected to the wider archipelago via both sea and air links and lies upwind from the prevailing easterlies which cross the archipelago. Together, these human aided and natural routes to mosquito dispersal could facilitate the passage of introduced diseases from San Cristóbal to other islands (Peck, 1994). Last, San Cristóbal was the first island settled in Galápagos and likely the first location avian pox was introduced (Parker et al., 2011). It is therefore home to one of the longest pox‐exposed communities of endemic bird species and could offer a baseline understanding of transmission dynamics in the archipelago.

The goal of this study was to survey the prevalence of diseases in passerine species on San Cristóbal, focusing on the established pathogens: avian haemosporidian parasites (including avian malaria parasites) and *Avipoxvirus*. Avian haemosporidian parasites include three genera of protists: *Leucocytozoon* spp. and the two avian malaria genera, *Plasmodium* and *Haemoproteus*. Haemosporidia require arthropod vectors for their transmission and can be lethal to susceptible bird species where high parasitemia results in anemia and damage to the liver and spleen (Palinauskas et al., 2008). *Avipoxvirus*, meanwhile, is transmitted through contact or indirectly through passive transport by arthropod vectors. This virus causes avian pox, which typically manifests as tumor‐like epidermal lesions, with severe cases resulting in permanent scaring and deformation of feet and beaks (Thiel et al., 2005). Both diseases are associated with decreased survival and reproduction in wild bird populations (Atkinson & LaPointe, 2009; Curry & Grant, 1989; Dadam et al., 2019; Lachish et al., 2012). While avian pox has been present in Galápagos for decades, the ongoing impact of this disease on endemic species is still poorly understood, while the introduction of a virulent malarial parasite could have devastating consequences (Levin, 2013).

**FIGURE 1:**
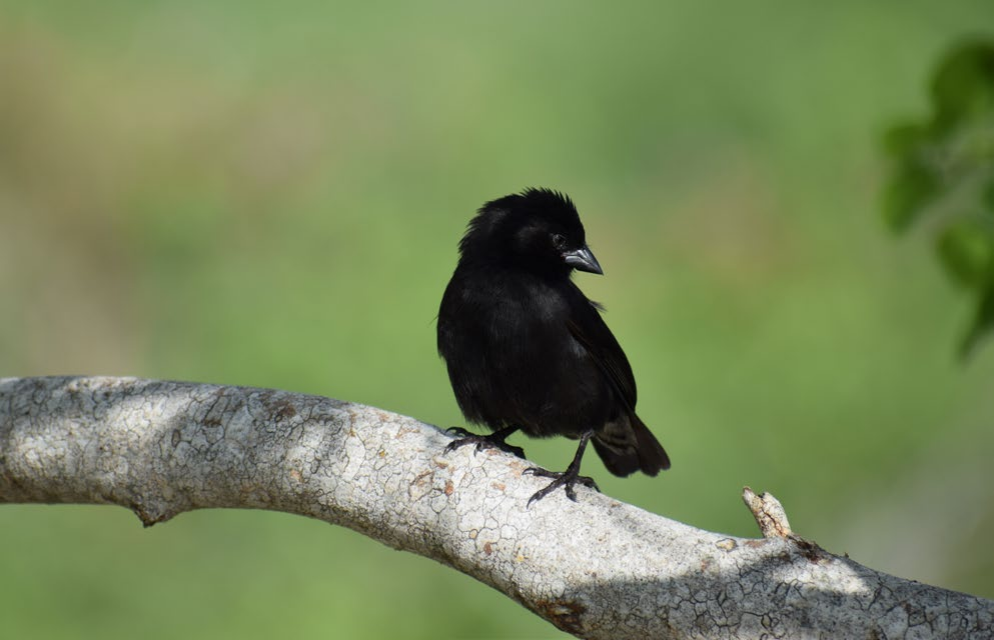
The small ground finch (*Geospiza fuliginosa*) – an iconic endemic Galápagoan bird species and the most numerous of the eleven species encountered in this study

To study these diseases, we caught passerines between 2016 and 2020 at rural and urban sites, including lowland and higher elevation localities. Our first aim was to identify whether avian malaria lineages detected elsewhere in the archipelago are currently in circulation on San Cristóbal, while also screening for novel lineages. Our second aim was to investigate the status and impact of avian pox infections on the island. To identify conditions favouring pox transmission, we tested for differences in prevalence between habitats and between years which presented variable climatic conditions. In the lowlands, urban areas have been associated with introduced populations of the disease vector *C*. *quinquefasciatus* by providing artificial breeding sites, while the availability of natural breeding sites in rural areas is more dependent on seasonal precipitation (Asigau et al., 2017; Whiteman et al., 2005). Highland areas on San Cristóbal are more humid and could provide more consistent habitat for vector populations, although this effect is likely vector specific (Asigau et al., 2017; Bataille et al., 2010), we therefore anticipated higher disease prevalence at urban and highland sites. In the Galápagos, high precipitation and warm conditions dominate December through April, during which land birds typically commence breeding (Grant & Boag, 1980). ENSO influences this seasonality, with El Niño phases associated with warmer sea temperatures and increased precipitation, while La Niña phases intensify arid conditions and can result in drought (Grant & Boag, 1980; Grant & Grant, 1987; Trueman & D’Ozouville, 2010). Yearly variation in temperature and precipitation in lowland areas, driven by the El Niño‐Southern Oscillation (ENSO), could therefore provide indication towards the relative role of vectors in disease transmission, as wetter years are associated with increased vector breeding activity and would likely lead to peaks in pox prevalence. Lastly, we tested for associations between pox infection and body condition in the small ground finch (*Geospiza fuliginosa*, Figure 1), the most commonly encountered host species.

## 2 METHODS

### 2.1 Capture and sampling

**FIGURE 2:**
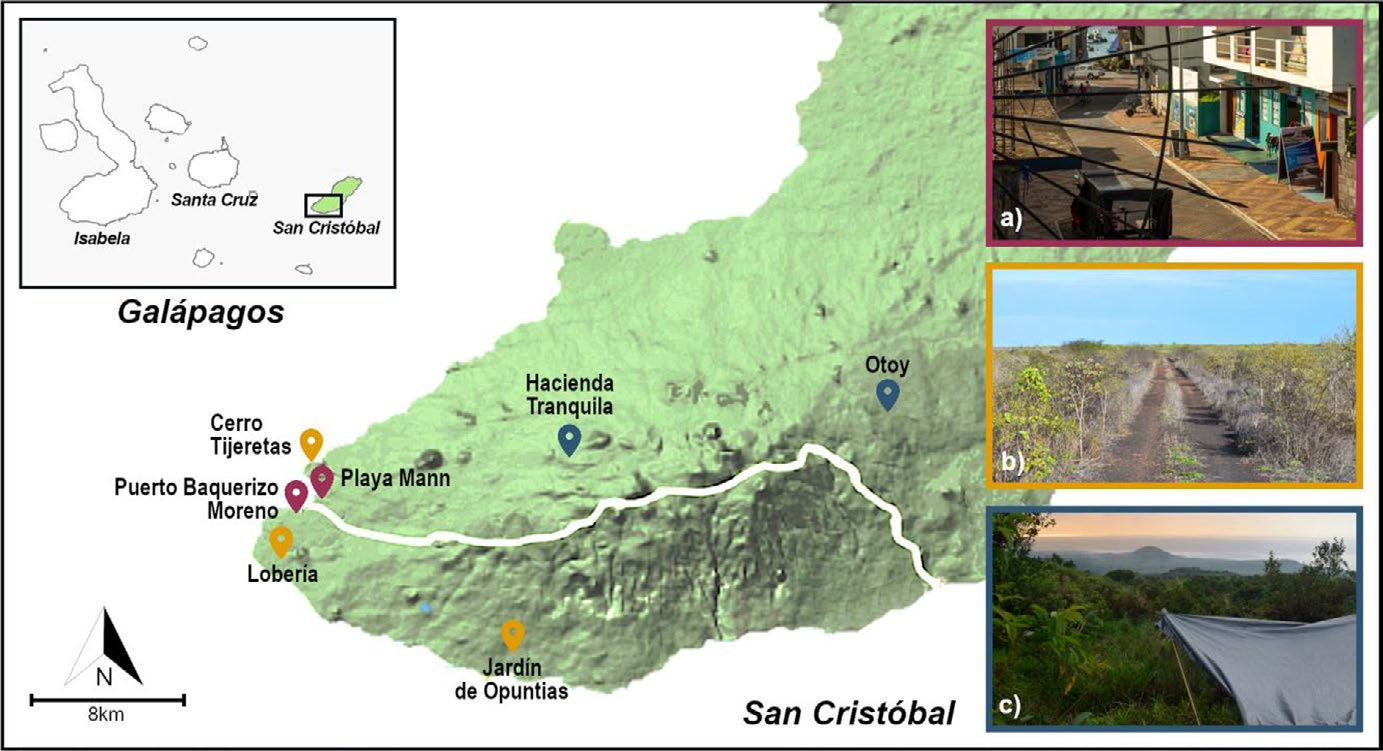
San Cristóbal Island with study site locations and habitat photos: (a) Playa Mann and Baquerizo are urban sites (in purple); (b) Cerro Tijeretas, Loberia, and Jardín de Opuntias are rural lowland sites (in gold); (c) Hacienda Tranquila and Otoy are highland rural sites (in blue). A white line indicates the main road which traverses the south of the island. Photo credit: (a) Dr. Kiyoko Gotanda

Birds were caught using mistnets on San Cristóbal between January and March during 2016, 2018, 2019, and 2020. Capture sites were classified as either highland (sampled in 2019 only), urban, or rural. Urban and rural sites were in the arid coastal lowlands (below an elevation of 50 m) and share a similar climate (~4 km apart). Rural and highland sites were situated at least 3 km away from urban areas (defined as the towns of Puerto Baquerizo Moreno and El Progreso). Highland sites were within the humid zone above 200 m of elevation with characteristicly higher precipitation than that of the rural lowland sites (Hamann, 1979; Trueman & D’Ozouville, 2010). Sites and capture numbers are summarized in Table 1 and Figure 2. Birds were fitted with unique ID bands, weighed, and measured (tarsus and wing length) on capture. Sex was determined from plumage characteristics (Price, 1984); ambiguous individuals were molecularly sexed (*N* = 341). Birds were aged as juveniles or as second year adults based on plumage and juvenile‐type gape flange. We recorded active pox lesions on bare skin, which are characteristically ulcerated and wart‐like in appearance and often present some bleeding or exudate (van Riper & Forrester, 2007). In the Galápagos, there are no other known diseases which result in these growths, although other potential causes have been noted (Kleindorfer & Dudaniec, 2006; Parker et al., 2011; Zylberberg et al., 2012). When possible, we collected swabs to broadly confirm etiology by identifying the specific pox strains circulating on San Cristóbal. As PCR detection of avipoxvirus from pox lesion swabs can have low sensitivity, we used our observations of pathology as representative of prevalence but caution that this prevalence can only be regarded as that of apparent pox prevalence. Therefore, mentions of pox throughout refer to pox‐like infection (Parker et al., 2011; Samuel et al., 2018; Williams et al., 2014). We also recorded signs of past pox infection, as evidenced by missing digits and scaring caused by lesions (Kleindorfer & Dudaniec, 2006; Zylberberg et al., 2012). Recording of pox in 2016 was not systematic, and estimates for this year are likely conservative. Blood samples were taken by brachial venipuncture in 2018 and 2019 and stored in 96%–100% ethanol. For a subset of blood samples, ~10 μl was used to make blood smears (*N* = 289). Slides were methanol fixed on the day of capture and later stained using Richard‐Allan Scientific™ Three Step Stain (Thermo Scientific™). Selected slides were scanned at low magnification (×200) for 5 min before the study of 100 fields at high magnification (×1000) under oil immersion using an Olympux BX61 microscope. Fieldwork was conducted under permit of the Galápagos National Park (GNP) (PC‐57‐15; PC‐36‐16; PC‐14‐17; PC‐03‐18; PC‐28‐19; & PC‐61‐20) and adhered to institutional ethics guidelines.

**TABLE 1:** Capture numbers per species and capture sites^a^ (locations shown in Figure 2) from the island of San Cristobal for 2016, 2018, 2019, and 2020 with numbers in parentheses indicating the subset of birds which presented evidence of pox (active pox lesions, past pox scarring)

	Rural lowlands									Urban lowlands					Highlands	
	Lobería				Tijeretas			Opuntias		Playa Mann			Baquerizo		Otoy	H.T.
	2016	2018	2019	2020	2016	2018	2019	2018	2019	2016	2018	2019	2018	2019	2019	2019
Small ground finch *Geospiza fuliginosa*	13 (2,0)	192 (1,4)	114 (7,6)	42 (2,10)	10	27	8	8	13	42 (4,0)	56	54 (5,6)	2	12 (1,1)	140 (3,6)	82 (2,11)
Medium ground finch *G*. *fortis*	3 (1,0)	13	55 (1,4)	4	3 (1,0)	9	2	–	1	2	24	21 (4,1)	–	9 (2,1)	3	7 (1,2)
Yellow warbler *Setophaga petechia aureola*	–	19	26	1	–	2	4	–	3 (0,1)	–	–	13	–	8 (1,0)	17 (1,0)	15 (1,0)
Small tree finch *Camarhynchus parvulus*	8 (1,0)	12	7 (1,0)	4	1	1	3	–	1	1	1	3	–	1	3	4
Galápagos flycatcher *Myiarchus magnirostris*	–	2	13 (1,0)	–	–	2	1	–	9	–	1 (1,0)	1	–	–	4	7 (0,1)
Other species^b^	–	1	–	–	–	–	–	9 (1,0)	5 (0,1)	–	–	3	–	1	12	5
Totals	24 (4,0)	239 (1,4)	215 (10,10)	51 (2,10)	14 (1,0)	41	18	17 (1,0)	32 (0,2)	45 (4,0)	82 (1,0)	95 (9,7)	2	31 (4,2)	179 (4,6)	120 (4,14)
Active pox %	16.7	0.4	4.7	3.9	7.1	–	–	5.9	–	8.9	1.2	9.5	–	12.9	2.2	3.3
Past pox indicators %	–	1.7	4.7	19.6	–	–	–	–	6.3	–	–	7.4	–	6.5	3.4	11.7
Total pox %	16.7	2.1	9.3	23.5	7.1	–	–	5.9	6.3	8.9	1.2	16.8	–	19.4	5.6	15

^a^ Sites abbreviated; Tijeretas = Cerro Tijeretas, Baquerizo = Puerto Baquerizo Moreno (locations within the town center), Opuntias = Jardín de Opuntias, and H.T. = Hacienda Tranquila.

^b^ Other species caught included: smooth‐billed ani (*Crotophaga ani*) (Otoy = 5, Playa Mann = 1), dark‐billed cuckoo (*Coccyzus melacoryphus*)(Otoy = 1), woodpecker finch (*Camarhynchus pallidus*) (Otoy = 3, Lobería = 1), grey warbler‐finch (*Certhidea fusca*) (Otoy = 3, H.T. = 3, Opuntias = 2 (1)), cactus finch (*Geospiza scandens*) (Opuntias = 11), and San Cristóbal mockingbird (*Mimus melanotis*) (Playa Mann = 4, Baquerizo = 1, Opuntias = 1, H.T. = 1).

### 2.2 Molecular methods

DNA was extracted from blood samples and pox swabs using DNeasy Blood & Tissue extraction kits (QIAGEN^®^). Sexing was performed using modified CHD1F‐CHD1R primers (Dobreva et al., 2021). To detect haemosporidian infections, we performed nested‐polymerase chain reactions (PCR); one specific to *Plasmodium*/*Haemoproteus* parasites and the other specific to *Leucocytozoon* (Hellgren et al., 2004). We screened a subset of highland birds for *Leucocytozoon* parasites as the highlands provide the only suitable habitat for their vectors (blackflies – *Simulium* spp., whose larvae develop in flowing streams (Abedraabo, 1992)). A PCR approach was also applied to detect *Avipoxvirus* from swabs (Lee & Lee, 1997). Amplicons were identified by gel electrophoresis and sequenced using either the primer HaemFL (for *Leucocytozoon* amplicons), HaemR2 (for Plasmodium/Haemoproteus amplicons), or bidirectionally for avipoxvirus amplicons through Eurofins sequencing (Eurofins‐MWG). Sequences were analyzed using Geneious (Geneious^®^ 9.1.5, Kearse et al., 2012) and identified to species or strain via BLAST on the NCBI database.

### 2.3 Statistical analysis

Statistical analyses were conducted using R version 3.5.1 (R Core Team, 2018) in RStudio v0.99.902 (RStudio Team, 2017). We tested predictors of pox infection using logistic regression (logit function), with infection status (binary) as the response variable. We first modelled the role of prevailing climatic conditions (El Niño or La Niña) in determining active pox prevalence using data collected in the lowlands (as highlands were only sampled in 2019); this dataset therefore included samples from 2016, 2018, 2019, and 2020 and was comprised of 891 individuals. We classified El Niño and La Niña years based on the ONI (Oceanic Niño Index) compiled and published by NOAA (National Oceanic and Atmospheric Administration; NOAA, 2020). Previous studies have highlighted the strong influence of ENSO on annual precipitation in the Galápagos (Trueman & d’Ozouville, 2010; Zhang et al., 2014). In these models, climate (El Niño or La Niña), host species (reduced to the species for which we had captures in each site class): medium ground finch (*Geospiza fortis*), small ground finch (*Geospiza fuliginosa)*, small tree finch (*Camarhynchus parvulus*), Galápagos flycatcher (*Myiarchus magnirostris*), San Cristóbal mockingbird (*Mimus melanotis*) and yellow warbler (*Setophaga petechial aureola*) and site class (urban or rural) were included as explanatory terms. We took a similar approach to model past pox infection in these same species, but instead included climate in the preceding year (El Niño or La Niña preceding) and site class as explanatory terms. This model did not use data from 2016 as past pox incidence was not recorded in that year, resulting in a dataset of 808 individuals.

We ran similar logistic regression models for pox data (active and past pox) collected only in 2019 (*N* = 670) to specifically explore variation in prevalence between sites. Explanatory terms were site (highland, urban, and rural) and host species (the same six species for which there were captures at all three site classifications). Candidate models were selected using the dredge function (MuMIn package; Bartoń, 2019) and reduced if the Akaike Information Criteria (AIC) estimator decreased by at least 2.0, improving model fit, otherwise the model with the fewest terms was selected (Zuur et al., 2009). A Pearson's chi‐squared test was applied to test the relationship between past pox and active pox incidence at our three site classes.

To explore variation in body condition in relation to pox infection we used data collected for small ground finch (the species for which we had most records of active pox infections). We calculated the scaled mass index derived from body mass (SMI) (Peig & Green, 2009). This condition index approach allows for an individual's current condition (i.e., body mass) to be scaled by their overall structural size (i.e., tarsus length). To obtain SMI values, we calculated the scaling exponent b_SMA_ which is the slope obtained from a standardized major axis (SMA) regression of ln‐transformed mass on tarsus length. We calculated *b*_SMA_ separately for our highland and lowland sites (as there was significant variation in the relationship between tarsus length and mass between these sites). Additionally, when calculating b_SMA_ for lowland sites, we excluded data from 2019 as this provided the most reliable estimates, with the strength of association (*r^2^* x 100) increasing from 6% (including 2019) to 14% (excluding 2019) (Peig & Green, 2010). This difference was caused by a considerable reduction in the strength of association between tarsus length and mass in 2019 (e.g., in 2018 the correlation between tarsus and mass was *r* = 0.36 (*t*_278_ = 6.42, *p* < .001), while in 2019 the correlation decreased to *r* = 0.17 (*t*_412_ = 3.52, *p* < .001). We also explored the relationship between pox infection and tarsus length (structural size) and between pox infection and wing length (more condition dependent than tarsus length; Green, 2001). As birds at highland sites were only caught in 2019, we analyzed the dataset in two parts; birds caught in 2019 across all study sites and those caught in the lowlands across all years. Linear mixed‐effects models were then applied using SMI^mass^, tarsus length, or wing length as response terms. Infection (presence or absence of active or past pox infection), day of capture, and sex were included as fixed effects in all models while site of capture was included as a random effect. Year of capture was included as a fixed effect in lowland models while tarsus length was included as a fixed effect in models where wing length was the response term. Additionally, an interaction term was included between infection status and day of capture to account for the varying impact infected birds might experience dependent on environmental variability (e.g., changes in rainfall). We used restricted maximum likelihood to estimate model parameters and calculated the marginal *R^2^* to assess final model fit (Nakagawa & Schielzeth, 2013). Model selection was carried out as previously described.

## 3 RESULTS

### 3.1 Site surveys

On San Cristobal, we caught 1205 birds across the four‐year study period, encompassing 11 species. Of these species, five accounted for 97% of our sampling; the small ground finch, medium ground finch, small tree finch, yellow warbler, and the Galápagos flycatcher (*Myiarchus magnirostris*) (Table 1). Capture success was higher at rural sites (i.e., rural lowlands and highlands) than at urban lowland sites (*N*_rural_ = 951 birds caught over 42 catching days versus *N*_urban_ = 255 caught over 28 days, excluding two birds for which capture date was unaccounted for). Highland birds were caught in 2019 (*N* = 350). Our sampling included three El Niño years (2016, 2019, and 2020) and one La Niña year (2018, which had been preceded by a La Niña year in 2017).

### 3.2 Characterizing pox infections

Of the 19 swab samples collected, 74% tested positive for Avipoxvirus. Negative results were obtained for five swabs, most likely reflecting poor acquisition or extraction of pox DNA as opposed to lesions being of a different etiology. We detected the two strains previously reported to be circulating in the Galápagos, both of which are classified as canarypox viruses (Thiel et al., 2005). Gal2 was the most prevalent strain at 79% (i.e., 11/14 classified infections), whereas Gal1 was detected in 29% of infections (i.e., 4/14 samples, in one case as a mixed infection with Gal2). This contrasts with previous sampling from Santa Cruz where Gal2 represented just 33% of infections vs. 77% Gal1 (Thiel et al., 2005). There was no discernible pattern to the geographical or host distribution of these strains, although the small sample size precluded any statistical test.

### 3.3 Disease prevalence

Of the birds caught, 995 were screened for the haemosporidian parasites *Plasmodium* and *Haemoproteus*, and 88 of the highland birds were also screened for *Leucocytozoon*. None were found to be positive for any of these targeted parasites, and no parasites were found from slide scans of these samples.

**FIGURE 3:**
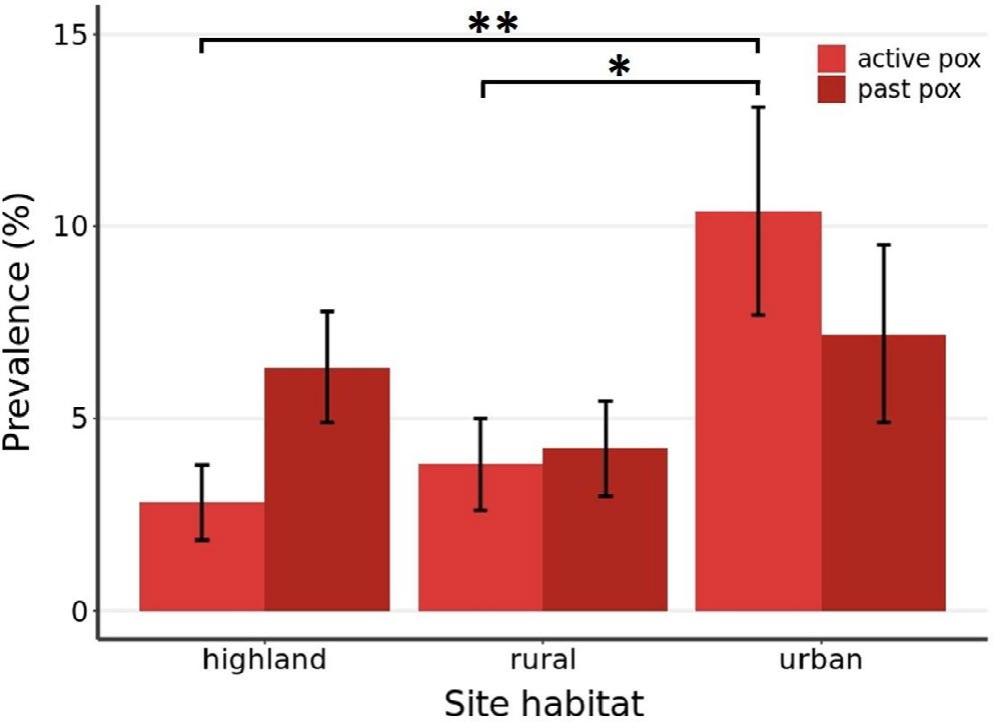
Predicted pox prevalence in San Cristóbal Island avifauna (medium ground finch, small ground finch, small tree finch, San Cristóbal mockingbird, Galápagos flycatcher, and yellow warbler) dependent on capture site in 2019. Prevalence data plotted from models of active pox and past pox. Significant post‐hoc Tukey test was used for differences between sites denoted by (*p < .05, **p < .01). No significant differences between sites for past pox prevalence as site was not found to be a significant predictor in the final model. Error bars show standard error

**FIGURE 4:**
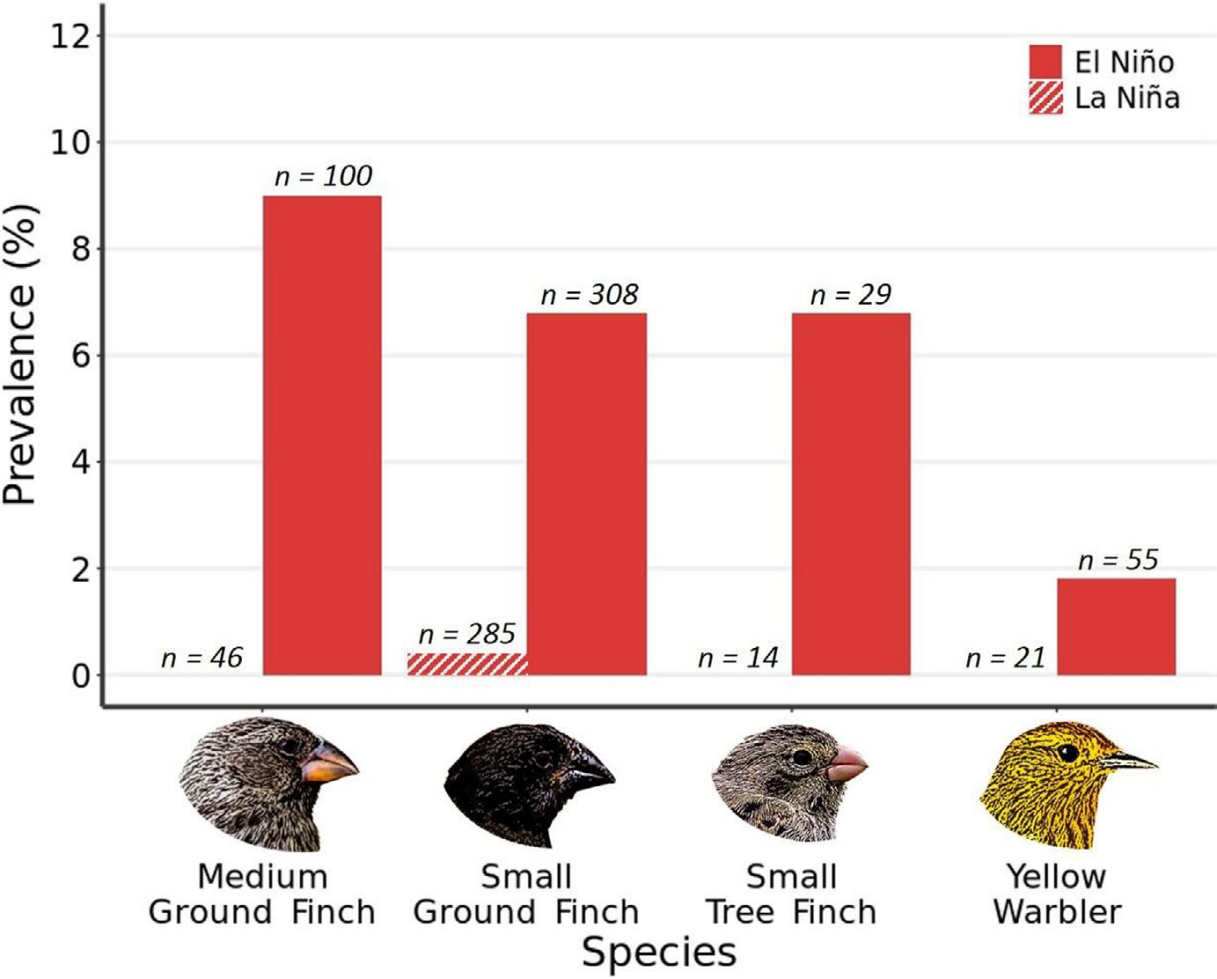
Active pox prevalence in San Cristóbal Island avifauna (four most common species) dependent on climatic conditions. Prevalence data plotted by species in years with El Niño climactic conditions or La Niña conditions. Numbers at the top of the bars indicate number of birds visually screened for pox (*n*)

In contrast, we found evidence of pox infection in 97 of the 1205 birds examined, with 45 displaying active lesions and 52 evidencing past pox infections. The prevalence of active pox (lesions reflecting current infection) was found to vary primarily with the prevailing climatic conditions and site of capture. Ninety‐four per cent of active pox infections were recorded in El Niño years, despite extensive sampling in the La Niña year of 2018 (*N* = 371 birds). This was not the result of a temporal trend towards higher prevalence of active pox infections as 25% of active pox cases were recorded in 2016 (Table 1). Prevalence also varied significantly between capture sites, with site retained in both models of active pox infection across years in the lowlands and in 2019 (Table 2 and Appendix S1). Indeed, when considering 2019 only (i.e., a year of high pox prevalence and extensive sampling, *N* = 670), site was a significant predictor of pox infection (*X^2^* = 9.9 [df = 2, 667], *p* = .007) and a post‐hoc Tukey test showed urban sites had a significantly higher pox prevalence than both rural (*p* = .04) and highland sites (*p* = .008; Appendix S1, Figure 3). Finally, we found no significant difference in active pox prevalence between host species with this term dropped from all simplified models, although recorded prevalence was generally higher in the ground finches (*Geospiza*) than the Yellow Warbler (Figure 4).

**TABLE 2:** Generalized linear model selection of the top five candidate models for active and past pox infection prevalence in response to whether the year had an El Niño climate (yes/no), or an El Niño climate in the preceding year (yes/no)

Response	Variables^a^	AICc	dAICc	df	Weight
Active Pox	El Niño & Site	276.8	0	3	0.78
	El Niño	280.1	3.28	2	0.15
	El Niño, Host Species & Site	281.8	5.01	8	0.06
	El Niño & Host Species	285.9	9.11	7	0.01
	Site	297.8	20.98	2	0
Past Pox	El Niño Preceding	260.5	0	2	0.45
	El Niño Preceding & Site	261	0.51	3	0.35
	El Niño Preceding & Host Species	263.2	2.69	7	0.12
	El Niño Preceding, Site & Species	264	3.52	8	0.08
	Intercept	277.7	17.2	1	0

Note

Site (urban or lowland) and host species were included as fixed effects. Final models (bold font) were selected on the basis of the lowest AICc in conjunction with the fewest term, dAICc is relative to the model with the lowest AICc score.

^a^ Host species included depended on the available data = medium ground finch, small ground finch, small tree finch, San Cristóbal mockingbird, Galápagos flycatcher,and yellow warbler. Models for active pox use data collected in all years but exclude highland sites which were only sampled in 2019. Models for past pox additionally exclude data from 2016 as past pox incidence was not recorded.

Prevalence of past pox infection was also influenced by climate (Table 2, Figure 4), with past pox incidence significantly higher in years where the preceding year had El Niño conditions (*X^2^* = 19.2 [df = 1, 806], *p* < .001) where the effect of a preceding El Niño increased the odds of past pox infection by a factor of 7.8 (95%CI: 3.3–17.1). Unlike active pox, past pox prevalence did not vary significantly between capture sites in 2019. As with active pox, host species was not retained in any of the top models. A chi‐squared test showed that the distribution of active and past pox infections within sites differed significantly (*X* ^2^ = 7.20, df = 2, *p* = .03), with active pox prevalence higher than past pox prevalence at urban sites while past pox prevalence was higher than active at highland sites (Figure 3).

### 3.4 Infection costs

**FIGURE 5:**
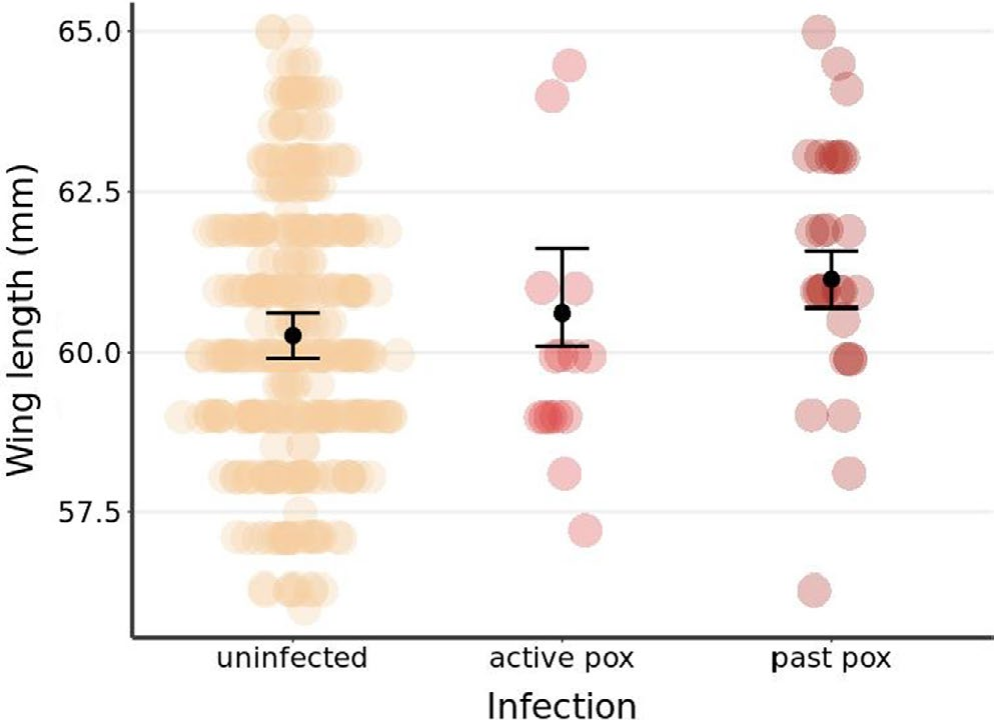
Wing length as predicted for small ground finches caught in 2019 dependent on infection. Black points and error bars are predicted length with standard error. Distribution of the raw data represented by underlayed points. Wing length was significantly longer in individuals with past pox when controlling for variation between site, and accounting for sex, tarsus length, and the day of capture (Appendix S2)

We found no relationship between pox infection and body condition (SMI) or tarsus length in small ground finch, as both active and past pox incidence were dropped from the minimal models during model selection (Appendix S2). Past pox infection was, however, significantly associated with a marginal increase in wing length (0.87 mm increase over non‐infected birds, *p* = .002) in 2019 (Figure 5, Table 3).

**TABLE 3:** Results of a linear mixed model explaining wing length in small ground finch in 2019 in relation to pox infection

Fixed Effect	Estimate	SE	df	*t*	*p*
Intercept	58.2	0.42	135	137.2	<.001
Infection					
Active Pox	0.35	0.38	391	0.9	.36
Past Pox	0.87	0.28	392	3.1	.002
Day	0.07	0.01	360	7.6	<.001
Sex (Male)	1.82	0.15	393	11.9	<.001
Tarsus	0.42	0.08	395	5.2	<.001

Note

Fitted random effect of capture site (intercept: SD = 0.97, residual: SD = 1.43), marginal *R* ^2^ = 0.38, conditional *R* ^2^ = 0.58, *p*‐values estimated via *t*‐tests using the Satterthwaite approximations to degrees of freedom.

## 4 DISCUSSION

Our survey of introduced diseases in the terrestrial avifauna of San Cristóbal, Galápagos, has updated knowledge of disease prevalence and possible emergence on this well‐connected island. First, we report no recent introductions of novel haemosporidian blood parasites despite extensive screening in lowland areas around human transit. Second, we found avian pox prevalence varies significantly with prevailing climate and site classification, with prevalence reaching a maximum of 11% in 2016 and in 2019 at urban sites. Identifying the factors (particularly abiotic) that drive among‐year variation in pox prevalence will further our understanding of pox transmission across the archipelago and has important implications for the conservation of endemic bird species.

Avian haemosporidia (*Plasmodium* and *Haemoproteus* lineages) have been previously reported across Galápagos (Jaramillo et al., 2017; Levin, 2013; Perlut et al., 2018). Sporadic detection of *Plasmodium spp*. in native species and more regular detection in migrants has highlighted the persistent threat of an introduction event of more virulent invasive haemosporidia into endemic bird populations (Perlut et al., 2018). These parasites may be introduced through any of the transport routes connecting San Cristóbal to the mainland and to other islands (Toral‐Granda et al., 2017). For example, an outbreak of avian malaria could be seeded by the transportation of an infected continental mosquito. Indeed, despite fumigation measures introduced on air transport, genetic analysis of *C*. *quinquefasciatus* populations has found clear evidence of repeated introductions to San Cristóbal (Bataille, 2009). With *C*. *quinquefasciatus* populations established on the islands, migrating bird species represent another route of entry for pathogens. Bobolinks (*Dolichonyx oryzivorus*), for example, occasionally migrate through the highlands of San Cristóbal (Perlut & Renfrew, 2016). Because these birds can arrive infected, the presence of a competent vector would be sufficient to transfer their parasites to endemic host species (Levin et al., 2013; Perlut et al., 2018). Despite these candidate introduction routes, we have found that virulent haemosporidian parasites (such as *P*. *relictum*) remain absent from San Cristóbal. Additionally, the absence in our samples of *Haemoproteus multipigmentatus*, an introduced haemosporidian circulating in the Galápagos Dove (*Zenaida galapagoensis*), reflects the decline of this bird species on the island (Dvorak et al., 2017, 2019; Jaramillo et al., 2017). Our results suggest that endemic birds of San Cristóbal are not significantly impacted by avian malaria, which may in part be due to the relatively recent establishment of *C*. *quinquefasciatus* (first reported in 1985, in contrast to Hawaii where introduction is thought to have occurred in the 1820s) (Warren, 1968; Whiteman et al., 2005). However, the risk of introduction is unabated, and without more extensive controls the continued surveillance of populations at high risk (i.e., those resident to areas with higher propability of exposure such as near airports and seaports) will be an essential conservation tool in response to the future emergence of invasive haemosporidia.

In contrast to avian malaria, avian pox has been established since introduction in the 1890s (Parker et al., 2011). On San Cristóbal, yearly variation in the probability of pox infection was substantial, ranging from <1% to 11% prevalence between years. Strikingly, this annual variation tracked the phases of the ENSO: El Niño and La Niña. We noted that 2016 and 2019 presented the highest prevalence of pox and corresponded to prevailing El Niño conditions, whereas weak La Niña conditions present in 2018 corresponded to pox prevalence falling to less than 1% incidence (NOAA, 2020). It appears likely that changes in climate (e.g., precipitation) are facilitating pox transmission and this may result from increased vector activity in wetter years (Arendt, 1985; Holmgren et al., 2001; O’Connor et al., 2010). Unlike avian malaria, which requires a susceptible vector host for development and transmission, avian pox can be theoretically vectored mechanically by any biting insect (van Riper & Forrester, 2007). This could include the now abundant invasive *P*. *downsi*; the larval stage of this species are nest parasites known to take blood meals from both parents and chicks within the nest and could therefore increase transmission between infected parents and offspring (Quiroga et al., 2020; Wiedenfeld et al., 2007). Transmission could also be amplified through increased contact events between infected birds during breeding and territory defense (Silk et al., 2014) and/or alternatively by host tradeoffs between breeding and immune investment (Bonneaud et al., 2003). We did not detect a progressive increase in pox prevalence across the four years of sampling, this contrasts with pox surveillance on Santa Cruz conducted at the start of the 21st century (Kleindorfer & Dudaniec, 2006). However, as El Niño weather events are modelled to increase in both frequency and intensity with climate warming (Trueman & D’Ozouville, 2010; Wang et al., 2017), more severe outbreaks of avian pox on San Cristóbal (and across the archipelago) could become increasingly common.

Urban sites hosted the highest prevalence of pox, suggesting a role of human development in facilitating this pathogen. Indeed, following introduction to the archipelago, *Avipoxvirus* was detected around settlements (Parker et al., 2011) and prevalence has been associated with human land use on Santa Cruz island (Zylberberg et al., 2013). While such a pattern could result from greater numbers of insect vectors at sites with human development, blood meal analysis suggests that both the endemic mosquito *Ae*. *taeniorhynchus* and *C*. *quinquefasciatus* preferentially feed on human blood where available (Asigau et al., 2019; Bataille et al., 2012). Birds foraging on anthropogenic food sources at urban sites could also increase contact between individuals and increase transmission opportunities. However, the low prevalence of pox across both urban and rural sites in the driest year (i.e., 2018) suggests that inter‐year variation in environmental conditions may play a greater role in shaping pox prevalence than anthropogenically aided transmisson. We recorded lower pox prevalence in the highlands of San Cristóbal than lowland sites; an elevational effect reported elsewhere (Fessl & Tebbich, 2002; Kleindorfer & Dudaniec, 2006). Whether this results from differences in vector abundances (lower abundance of *Ae*. *taeniorhynchus* was recorded at higher inland elevations on Santa Cruz (Asigau et al., 2019)), differences in the environmental persistence of the virus itself (Rheinbaben et al., 2007), or due to differences in intraspecific population susceptibilities (Zylberberg et al., 2012) is yet to be determined.

With many endemic bird species in decline, determining the impact of increased pox prevalence remains a conservation priority (Dvorak et al., 2012). Previous studies have illustrated the potential cost of pox infection for host survival which, given interspecific differences in prevalence, is likely to vary between host species (Curry & Grant, 1989; Kleindorfer & Dudaniec, 2006; Vargas, 1987; Zylberberg et al., 2012, 2013). Host species in other avian communities (e.g., such as those in Hawaii) have been found to present differences in susceptibility to pox, with non‐native species having lower infection rates (Atkinson et al., 2005; Van Riper et al., 2002). We might have therefore predicted to find lower pox prevalence in the native subspecies of yellow warbler, a species which colonized the islands more recently than the endemic finches (Chaves et al., 2012; Lamichhaney, 2015). However, although pox prevalence was generally lower in yellow warblers, we found no evidence for significant variation in pox incidence between hosts species. This was also in contrast to prevalence patterns reported on Santa Cruz, where less pox was observed in tree finches (*Camarhynchus* spp.) than ground finches (*Geospiza* spp.) (Kleindorfer & Dudaniec, 2006). We caution that this finding may be influenced by the low capture numbers we obtained for some host species. More research is required to identify the degree to which pox suseptibility varries between host species in the Galápagos, a task which will help shed light on the future threat pox poses across the endemic avifauna.

On San Cristóbal, we found no relationship between current pox infection and body condition in small ground finches. While this finding should be treated with caution (due to the low incidence of pox positive birds sampled), it does support results from elsewhere in the archipelago (Zylberberg et al., 2012, 2013). Our finding that previously infected small ground finches tended to have longer wing lengths is difficult to interpret without additional data. It could indicate a selective effect of pox infection (whereby birds surviving infection were in better condition or better able to avoid predation), or result if birds which had previous infections were less likely to reproduce and instead invested in molt (Kleindorfer & Dudaniec, 2006; Snow, 1966). Both interpretations suggest avian pox carries a cost to the small ground finch, either through mortality or lost reproductive opportunities. Future studies could focus on more sensitive measures of condition (e.g., molecular indicators) and establishing health baselines for endemic species would aid in understanding the impact of current and future disease outbreaks (Stevenson & Woods, 2006).

To date, no study in Galápagos has comprehensively tracked the impact of avian pox on long term fitness and population stability, and little is known regarding the genetics underlying host immunity or resistance to the virus (Zylberberg et al., 2012). Questions also remain over the transmission dynamics of the two circulating canarypox strains, which appear to differ in frequency between islands (or perhaps year), and further still the role of vector mediated transmission versus environmental. This study provides an update to our current knowledge of two key disease threats on the second most populous island in Galápagos, and, in doing so, we have highlighted the knowledge gaps which remain ahead.

## CONFLICT OF INTEREST

The authors declare that there is no conflict of interest.

## AUTHOR CONTRIBUTIONS

**Joshua G. Lynton‐Jenkins:** Data curation (lead); Formal analysis (lead); Funding acquisition (supporting); Investigation (lead); Methodology (equal); Resources (supporting); Visualization (lead); Writing – original draft (lead); Writing – review & editing (supporting). **Andrew F. Russell:** Formal analysis (supporting); Investigation (supporting); Methodology (equal); Supervision (equal); Writing – original draft (supporting); Writing – review & editing (supporting). **Jaime Chaves:** Conceptualization (equal); Data curation (supporting); Funding acquisition (equal); Investigation (supporting); Methodology (equal); Project administration (lead); Resources (equal); Supervision (equal); Writing – review & editing (equal). **Camille Bonneaud:** Conceptualization (equal); Investigation (supporting); Methodology (equal); Project administration (equal); Resources (equal); Supervision (equal); Writing – original draft (supporting); Writing – review & editing (equal).

## Supporting information

Appendix S1‐S2

## Data Availability

Data supporting this study are openly available via Dryad (https://doi.org/10.5061/dryad.kwh70rz4z). Sequences are available on GenBank (OL634783‐95).
