# Supplementary material for: Avian disease surveillance on the island of San Cristóbal, Galápagos
Source: Ecol Evol. 2021 Dec 6;11(24):18422–33. doi: 10.1002/ece3.8431 (PMC8717262; doi:10.1002/ece3.8431)
Supplement: Supplementary file 1 — Appendix S1‐S2 [file ECE3-11-18422-s001.docx]

**Title:**  Avian disease surveillance on the island of San Cristóbal, Galápagos.

**Appendix S1.** Generalized linear model selection of the top 5 candidate models for active and past pox infection prevalence in response to site (urban, lowland rural or highland) and host species (Medium Ground Finch, Small Ground Finch, Small Tree Finch, San Cristóbal Mockingbird, Galápagos Flycatcher, & Yellow Warbler). Models use data collected in 2019, include highland sites and therefore focus on site variation in pox prevalence. Final models (bold font) were selected on the basis of the lowest AIC in conjunction with the fewest term, if the reduction in AICc was less than 2 all top models are displayed in (bold italics). dAICc is relative to the model with the lowest AICc score.

| Response | Variables | AICc | dAICc | df | Weight |
| --- | --- | --- | --- | --- | --- |
| **Active Pox** | **Site** | **247.2** | **0** | **3** | **0.93** |
|  | Intercept | 253.1 | 5.88 | 1 | 0.05 |
|  | Site & Host Species | 255 | 7.75 | 8 | 0.03 |
|  | Host Species | 259.7 | 12.45 | 6 | 0.00 |
|  |  |  |  |  |  |
| **Past Pox** | ***Host Species*** | ***293.7*** | ***0*** | ***6*** | ***0.40*** |
|  | ***Intercept*** | ***293.9*** | ***0.23*** | ***1*** | ***0.35*** |
|  | Host Species & Site | 295.9 | 2.21 | 8 | 0.13 |
|  | Site | 296.1 | 2.39 | 3 | 0.12 |
|  |  |  |  |  |  |

**Appendix S2**. Linear model selection of top 5 candidate models for species scale mass index (SMI), wing length, and tarsus length, in response to pox infection (active pox, past pox, or uninfected). a) Models used data from only lowland sites as highlands were only sampled in 2019 and additionally included sex, site (urban or rural), species (Medium Ground Finch and Small Ground Finch) and the presence of El Niño (yes/no) as explanatory terms. b) Models use data collected in 2019, an El Niño year, for G. fuliginosa (N = 404) and included highland sites. Final models (bold font) were selected on the basis of the lowest AICc in conjunction with the fewest term, if the reduction in AICc was less than 2 top models are displayed in bold italics with simplest model listed first, dAICc relative to model with the lowest AICc score.

| **Response** | **Variables** | **AICc** | **dAICc** | **df** | **Weight** |
| --- | --- | --- | --- | --- | --- |
| **a)** |  |  |  |  |  |
| **SMI** | **Year & (1\|Site)** | **2456.5** | **0** | **6** | **0.68** |
|  | Infection, Year & (1\|Site) | 2458.9 | 2.4 | 8 | 0.21 |
|  | Year, Day & (1\|Site) | 2460.9 | 4.34 | 7 | 0.08 |
|  | Infection, Day, Year & (1\|Site) | 2463.5 | 6.96 | 9 | 0.02 |
|  | Day & (1\|Site) | 2465.5 | 9.02 | 4 | 0.01 |
|  |  |  |  |  |  |
| **Tarsus** | **Sex, Year & (1\|Site)** | **1362.4** | **0** | **7** | **0.86** |
|  | Sex & (1\|Site) | 1367.2 | 4.73 | 4 | 0.08 |
|  | Infection, Sex, Year & (1\|Site) | 1368.5 | 6.03 | 9 | 0.04 |
|  | Infection, Sex & (1\|Site) | 1371.1 | 8.65 | 6 | 0.01 |
|  | Day, Sex, Year & (1\|Site) | 1373.7 | 11.27 | 8 | 0 |
|  |  |  |  |  |  |
| **Wing** | ***Infection, Sex, Tarsus, Year, Day & (1\|Site)*** | **2146.4** | **0** | **11** | **0.82** |
|  | Sex, Tarsus, Year, Day & (1\|Site) | 2150.6 | 4.11 | 9 | 0.11 |
|  | Infection, Sex, Tarsus, Year & (1\|Site) | 2151.3 | 4.86 | 10 | 0.07 |
|  | Sex, Tarsus, Year & (1\|Site) | 2156.8 | 10.31 | 8 | 0.01 |
|  | Infection * Day, Sex, Tarsus, Year & (1\|Site) | 2159.3 | 12.84 | 13 | 0 |
|  |  |  |  |  |  |
| **b)** |  |  |  |  |  |
| **SMI** | ***Intercept & (1\|Site)*** | ***1713.4*** | ***0*** | ***3*** | ***0.55*** |
|  | ***Sex & (1\|Site)*** | ***1715.3*** | ***1.9*** | ***4*** | ***0.21*** |
|  | Infection & (1\|Site) | 1716 | 2.58 | 5 | 0.15 |
|  | Infection, Sex & (1\|Site) | 1717.9 | 4.48 | 6 | 0.06 |
|  | Day & (1\|Site) | 1720.9 | 7.51 | 4 | 0.01 |
|  |  |  |  |  |  |
| **Tarsus** | **Sex & (1\|Site)** | **898.3** | **0** | **4** | **0.94** |
|  | Infection, Sex & (1\|Site) | 904.6 | 6.22 | 6 | 0.04 |
|  | Sex, Day & (1\|Site) | 906.2 | 7.83 | 5 | 0.02 |
|  | Infection, Sex, Day & (1\|Site) | 912.2 | 13.86 | 7 | 0 |
|  | Infection, * Day, Sex & (1\|Site) | 926.9 | 28.56 | 9 | 0 |
|  |  |  |  |  |  |
| **Wing** | **Infection, Sex, Tarsus, Day & (1\|Site)** | **1480.3** | **0** | **8** | **0.92** |
|  | *Sex, Tarsus, Day & (1\|Site)* | 1485.1 | 4.85 | 6 | 0.08 |
|  | Infection * Day, Sex, Tarsus & (1\|Site) | 1493.7 | 13.43 | 10 | 0 |
|  | Infection, Day, Sex & (1\|Site) | 1501.6 | 21.26 | 7 | 0 |
|  | Day, Sex & (1\|Site) | 1506.6 | 26.33 | 5 | 0 |
|  |  |  |  |  |  |
